# Salviolone from *Salvia miltiorrhiza* Roots Impairs Cell Cycle Progression, Colony Formation, and Metalloproteinase-2 Activity in A375 Melanoma Cells: Involvement of P21(Cip1/Waf1) Expression and STAT3 Phosphorylation

**DOI:** 10.3390/ijms23031121

**Published:** 2022-01-20

**Authors:** Valentina Zanrè, Rachele Campagnari, Antonietta Cerulli, Milena Masullo, Alessia Cardile, Sonia Piacente, Marta Menegazzi

**Affiliations:** 1 Department of Neurosciences, Biomedicine and Movement Sciences, Biochemistry Section, University of Verona, Strada Le Grazie, 8, I-37134 Verona, Italy; valentina.zanre@univr.it (V.Z.); rachele.campagnari@univr.it (R.C.); alessia.cardile@studenti.univr.it (A.C.); 2 Department of Pharmacy, University of Salerno, via Giovanni Paolo II n.132, I-84084 Fisciano, Italy; acerulli@unisa.it (A.C.); mmasullo@unisa.it (M.M.)

**Keywords:** cryptotanshinone, tanshinone IIA, diterpenoids, Cdk2, cyclin A2, retinoblastoma protein, P53, Akt, ERK1/2

## Abstract

Melanoma is a highly malignant solid tumor characterized by an elevated growth and propagation rate. Since, often, melanoma treatment cannot prevent recurrences and the appearance of metastasis, new anti-melanoma agents need to be discovered. *Salvia miltiorrhiza* roots are a source of diterpenoid derivatives, natural compounds with several biological activities, including antiproliferative and anticancer effects. Seven diterpenoid derivatives were purified from *S.* *miltiorrhiza* roots and identified by NMR and MS analysis. Tanshinone IIA and cryptotanshinone were detected as the main components of *S.* *miltiorrhiza* root ethanol extract. Although their antitumor activity is already known, they have been confirmed to induce a reduction in A375 and MeWo melanoma cell growth. Likewise, salviolone has been shown to impair the viability of melanoma cells without affecting the growth of normal melanocytes. The underlying anticancer activity of salviolone has been investigated and compared to that of cryptotanshinone in A375 cells, showing an increased P21 protein expression in a P53-dependent manner. In that way, salviolone, even more than cryptotanshinone, displays a multitarget effect on cell-cycle-related proteins. Besides, it modulates the phosphorylation level of the signal transducer and activator of transcription (STAT)3. Unexpectedly, salviolone and cryptotanshinone induce sustained activation of the extracellular signal-regulated kinases (ERK)1/2 and the protein kinase B (Akt). However, the blockage of ERK1/2 or Akt activities suggests that kinase activation does not hinder their ability to inhibit A375 cell growth. Finally, salviolone and cryptotanshinone inhibit to a comparable extent some crucial malignancy features of A375 melanoma cells, such as colony formation in soft agar and metalloproteinase-2 activity. In conclusion, it has been shown for the first time that salviolone, harboring a different molecular structure than tanshinone IIA and cryptotanshinone, exhibits a pleiotropic effect against melanoma by hampering cell cycle progression, STAT3 signaling, and malignant phenotype of A375 melanoma cells.

## 1. Introduction

Malignant melanoma is the most severe type of skin cancer for its high metastatic potential and elevated resistance to therapy [1]. To improve long-term survival in melanoma, the identification of new anti-tumor agents from plants is an attractive strategy. Remarkably, natural antitumor agents show a broad spectrum of mechanisms to hinder cancer progression, as they decrease the proliferation rate of malignant cells, diminish invasiveness and neo-angiogenesis, and generally display lower side effects in comparison with conventional antitumor drugs [2].

*Salvia* is the largest genus of the Lamiaceae family, from which many species excel in traditional medicine for the curative properties of their bioactive components [3]. *Salvia miltiorrhiza* Bunge, known as Danshen, is considered as one of the most important plants in traditional Chinese medicine [3], with a long history as a medicine as well as a healthy food.

Chemical investigations have shown that *S. miltiorrhiza* contains a large number of diterpenes, including various tanshinone analogues, phenolic compounds (including salvianolic acids and flavonoids), along with triterpenes. Tanshinones are phenolic diterpene compounds characterized by a phenanthrenequinone core extracted from a lipid-soluble fraction of the roots [4]. More than 40 tanshinones, including ortho-quinones (i.e., tanshinone I, tanshinone IIA, dihydrotanshinone, and cryptotanshinone) and para-quinones (i.e., isotanshinone I, isotanshinone IIA, and isocryptotanshinone), have been isolated from *S. miltiorrhiza* roots [4,5]. Tanshinone derivatives have attracted the attention of medicinal chemists and clinicians for their anticancer properties [6,7]. Many studies have shown that tanshinones can be used to treat solid tumors and hematological malignancies, as well as to protect against cardiovascular and cerebrovascular diseases [8,9,10,11]. A structure–activity relationship between different tanshinones has also been considered [12].

Multiple signaling pathways, including antiproliferative, anti-inflammatory, and antioxidative stress pathways, are involved in their actions as anti-cancer agents [11]. Among the large variety of diterpene quinones studied, tanshinone IIA and cryptotanshinone exhibit the most potent cytotoxic and anti-proliferative effects against many human cancer cells [7,13,14]. Indeed, tanshinone IIA induced endoplasmic reticulum stress, finally triggering apoptosis [15]. In contrast, cryptotanshinone has been reported to inhibit many crucial signaling pathways activated in cancer, among them the signal transducer and activator of transcription (STAT)3 in both renal and tongue squamous cell carcinoma [16,17].

In the past decade, several studies investigated the anti-melanoma effects of tanshinones. Li et al. reported that tanshinone IIA inhibits melanoma progression, migration, and invasion and induces autophagic cell death [18]. In contrast, Chen et al. [19] focused on cryptotanshinone action in two mouse melanoma cell lines. They found that cryptotanshinone blocks melanoma cell cycle progression, although it is unable to induce cytotoxic effects. Conversely, Ye et al. [20], besides a cytostatic effect, reported the cryptotanshinone ability to induce an apoptotic pathway in three human melanoma cell lines. They also demonstrated the cryptotanshinone potential to reduce cell migration, invasion, and colony formation [20], suggesting an anti-metastatic action of this natural product, as well. More recently, an in silico study suggested that cryptotanshinone could affect both nuclear factor kappa B (NF-ҝB) and STAT3 signaling pathways in melanoma [21]. Certainly, NF-ҝB and STAT3 play a pivotal role in oncogenesis because they trigger pro-survival signals in many cancer types, including melanoma [22,23].

**Figure 1:**
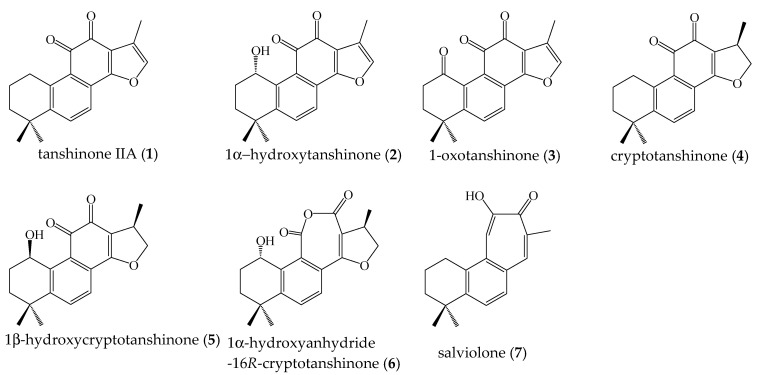
Compounds isolated from *S. miltiorrhiza* roots.

Herein, the phytochemical investigation of the ethanol extract of *Salvia miltiorrhiza* roots afforded seven compounds (**1**–**7**) (see Figure 1), whose structures were established by the extensive use of 1D and 2D NMR experiments as tanshinone derivatives (**1**–**6**) and a benzotropolone-type derivative (**7**). Moreover, the quantitative determination of compounds **1**–**7** in the extract of *S. miltiorrhiza* roots was carried out by an analytical approach based on LC-ESI/QTrap/MS, using a sensitive and selective mass tandem experiment called multiple reaction monitoring (MRM).

We assessed their ability to inhibit the viability of A375 and MeWo human melanoma cell lines, and we evaluated their structure–activity relationship. Besides the previously mentioned tanshinones IIA (**1**) and cryptotanshinone (**4**), we identified salviolone (**7**) as an active molecule against melanoma cells. Therefore, we comprehensively investigated on the A375 cell line the salviolone (**7**) anti-melanoma action in comparison to the more known cryptotanshinone (**4**) and we identified its principal molecular mechanisms.

## 2. Results

### 2.1. Isolation of Compounds **1**–**7** from Salvia miltiorrhiza Roots

The ethanol extract of *S. miltiorrhiza* roots was fractionated on a Sephadex LH-20 column, and the fractions were purified by semipreparative HPLC to afford compounds **1**–**7** (Figure 1), the structures of which were unambiguously elucidated by NMR spectroscopy (Figures S1–S7; Table S1). In this way, secondary metabolites isolated from *S. miltiorrhiza* roots were determined as diterpenoid compounds tanshinone IIA (**1**), 1α-hydroxytanshinone (**2**), 1-oxotanshinone (**3**), cryptotanshinone (**4**), 1*β*-hydroxycryptotanshinone (**5**), 1α-hydroxyanhydride-16*R*-cryptotanshinone (**6**), and salviolone (**7**).

### 2.2. Quantitative Analysis of Compounds **1–7** in S. miltiorrhiza Ethanol Extract

To determine the amount of diterpenoids **1**–**7** in *S. miltiorrhiza* ethanol extract, an LC-ESI/QTrap/MS/MS analysis, using MRM mode, was carried out. Multiple reaction monitoring (MRM) is a tandem mass spectrometric technique in which a specific transition from a precursor ion to a product ion is monitored for each compound, assuring a high selectivity and sensitivity [24]. MRM transitions (Table 1) were chosen based on the fragmentation pattern shown by each metabolite.

Tanshinone IIA (**1**) and 1α-hydroxytanshinone (**2**) showed pseudomolecular ions [M+H]^+^ at *m/z* 295 and 311, respectively. They were characterized by the same MS/MS fragmentation pattern originating from the main fragment ion [M+H−H_2_O]^+^, due to the neutral loss of water; thus this fragment ion was chosen for MRM analysis. For 1-oxotanshinone (**3**), the ion [M+H−CO]^+^ at *m/z* 281 was the predominant fragment ion obtained from the pseudomolecular ion at *m/z* 309. Thus, this fragment ion was chosen for MRM analysis. Cryptotanshinone (**4**) and 1*β*-hydroxycryptotanshinone (**5**) displayed pseudomolecular ions [M+H]^+^ at *m/z* 297 and 313, respectively. They were characterized by a key fragment ion [M+H-H_2_O-CO]^+^ at *m/z* 251 and 267, respectively, due to successive losses of H_2_O and CO. 1*α*-hydroxyanhydride-16*R*-cryptotanshinone (**6**) with the pseudomolecular ion [M+H]^+^ at *m/z* 329 displayed the main product ion at *m/z* 267, which was selected for MRM analysis. Finally, the fragmentation pattern of salviolone (**7**) was characterized by the neutral loss of water, producing an intense fragment ion [M+H-H_2_O]^+^ at *m/z* 251.

Based on the transitions selected for MRM experiments, the amount (mg/100 g) of each selected compound in *S. miltiorrhiza* roots was determined. Compounds **1**–**7** are present in the concentration range of 20.44–1379.00 (mg/100 g).

### 2.3. A375 and MeWo Melanoma Cell Viability was Affected by Tanshinone IIA, Cryptotanshinone, and Salviolone Extracted from Salvia miltiorrhiza Roots

Anti-melanoma activity of tanshinone IIA (**1**) and cryptotanshinone (**4**) has already been reported by several authors [18,19,20,25]. To attest the ability of compounds purified from *S.* *miltiorrhiza* to affect A375 and MeWo cell viability, we compared the activity of the more studied tanshinone IIA (**1**) and cryptotanshinone (**4**) with those of 1α-hydroxytanshinone (**2**), 1-oxotanshinone (**3**), 1*β*-hydroxycryptotanshinone (**5**), 1α-hydroxyanhydride-16*R*-cryptotanshinone (**6**), and salviolone (**7**). For tanshinone IIA (**1**) and cryptotanshinone (**4**), we used a range of concentrations deducted from the literature data, while for the other compounds, concentrations in a range of 5–50 µM were chosen from preliminary results.

In preliminary experiments, the evaluation of cell viability at different time after compound administration was carried out with sulforhodamine B (SRB) assay. Indeed, after 24-, 48-, and 72-h treatment, an evident time dependence in the inhibition of cell growth was registered (data not shown). Thus, to evaluate A375 and MeWo cell viability, we decided to perform further SRB experiments with each compound only after 72 h.

Our results showed a remarkable ability of tanshinone IIA to inhibit melanoma cell viability in a low micromolar range of concentration.

**Figure 2:**
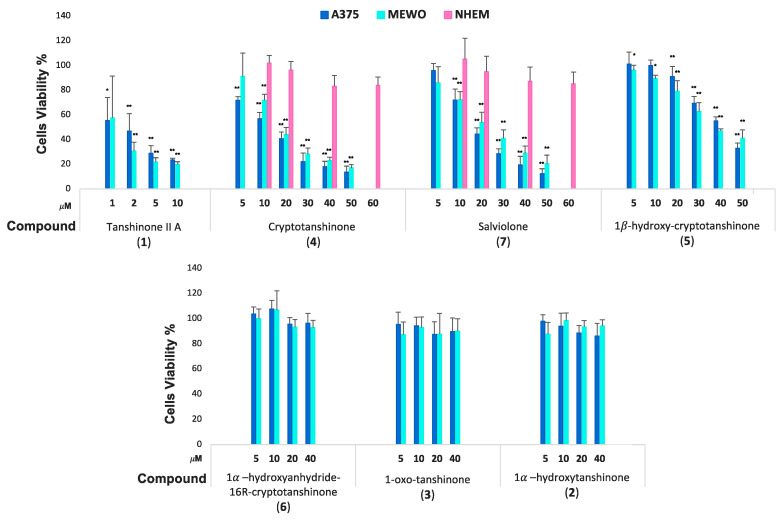
SRB cell viability assay in A375, MeWo melanoma cells, and NHEM cells in the presence of diterpene compounds. SRB viability assay of MeWo (light blue) and A375 (blue) melanoma cell lines with compounds **1**–**7**. SRB viability assay of the normal human epithelial melanocyte (NHEM) cell line (pink) with compounds **4** and **7**. Data acquired calculating the average ± SD of values obtained from three to four independent experiments were compared to data from non-treated control (* *p* < 0.05; ** *p* < 0.01).

The half maximal effective concentration (EC_50_) able to reduce cell viability in the A375 melanoma cell line was about 1 µM for tanshinone IIA (**1**), 14 µM for cryptotanshinone (**4**), 17 µM for salviolone (**7**), and 42 µM for 1*β*-hydroxycryptotanshinone (**5**) (Figure 2). Instead, in the case of MeWo cell line, the EC_50_ useful in diminishing cell viability was 2 µM for (**1**), 17 µM for (**4**), 22 µM for (**7**), and 45 µM for (**5**) (Figure 2). For its high EC_50_ value, compound **5** has been considered poorly active and so its mode of action has not been further investigated. However, 1-oxotanshinone IIA (**3**), 1-hydroxytanshinone (**2**), and 1α -hydroxy-anhydride-16*R* cryptotanshinone (**6**) were found to be ineffective at least until the concentration of 40 µM (Figure 2).

Thereafter, we decided to deepen the study of salviolone due to the novelty of its effect. The activities of salviolone on A375 melanoma cells have been compared with those of the well-studied cryptotanshinone. A375 cells were treated with the same concentrations of either salviolone or cryptotanshinone for their similarity in the EC_50_ values.

### 2.4. Salviolone and Cryptotanshinone Did Not Affect NHEM Cell Viability

To investigate whether salviolone (**7**) in comparison with cryptotanshinone (**4**) can inhibit normal human epithelial melanocyte growth, the effect of increasing concentrations of the compounds **7** and **4** on cell viability was measured by an SRB assay. Salviolone and cryptotanshinone did not affect NHEM viability at 10–20 µM, the concentrations near the EC_50_ values found for both compounds in A375 and MeWo melanoma cell lines. Higher concentrations (40–60 µM) of salviolone (**7**) or cryptotanshinone (**4**) can reduce NHEM viability by only 20% compared to 70–80% of viability reduction in melanoma cells at the same concentrations (Figure 2). Data suggest that salviolone (**7**) as well as cryptotanshinone (**4**) inhibit cell growth with higher selectivity for melanoma cells than normal melanocytes.

Therefore, we decided to investigate the molecular mechanisms underlying the anti-melanoma effect of salviolone (**7**) in comparison to the best-studied cryptotanshinone (**4**).

### 2.5. Signaling Pathways Involved in the Salviolone and Cryptotanshinone Anti-Melanoma Activity

To elucidate the mechanisms underlying the **7**- and **4**-induced cell growth inhibition, several signaling pathways involved in malignant melanoma A375 cell growth have been investigated.

#### 2.5.1. Salviolone and Cryptotanshinone Affect A375 Cell Cycle Progression

The downregulation of crucial cell-cycle-related proteins could explain the inhibition of melanoma cell viability by the active compounds.

Progression through the cell cycle requires cyclin-dependent kinase (Cdks) activation, hyper-phosphorylation of retinoblastoma protein (pRb), and sequential expression of cyclins [26].

**Figure 3:**
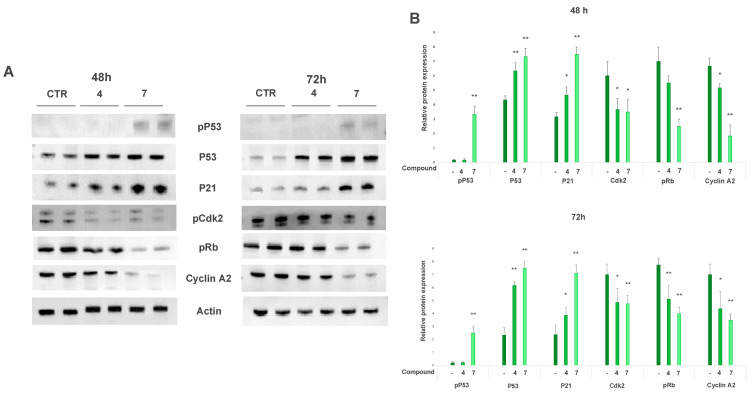
Cryptotanshinone (**4**) and salviolone (**7**) effect on the protein expression involved in cell cycle progression and pro-survival signaling. A375 melanoma cells were cultured in the presence (20 µM) or absence of salviolone (**7**) or cryptotanshinone (**4**) for 48 and 72 h. (**A**) Representative immunoblot showing the expression level of cell-cycle-related proteins; (**B**) histograms show the mean values ± SD of the protein expression level measured by densitometry deriving from three to four independent experiments. All comparisons were performed vs. each control sample after normalization with *β*-actin expression, * *p* < 0.05; ** *p* < 0.01.

The A375 cell line treated for 48 or 72 h with 20 µM cryptotanshinone (**4**) slightly reduced the expression of the active forms of Cdk2 (pCdk2) and cyclin A2, as well as the phosphorylation of Rb (Figure 3). Salviolone (**7**) more powerfully affected cell-cycle-related protein expression than cryptotanshinone (**4**). Indeed, 20 µM salviolone (**7**) strongly reduced pRb, pCdk2, and cyclin A2 expression levels in A375 cells treated for 48 or 72 h (Figure 3).

To identify the pathways involved in the inhibition of these cell-cycle-related proteins, we measured the expression of the Cdk-inhibitor P21(Cip1/Waf1). P21 can hamper the course of the cell cycle by binding Cdks and blocking their activity [27].

After 48 and 72 h, the P21 expression level significantly increased in A375 cells treated with 20 µM cryptotanshinone (**4**) and strongly increased with salviolone (**7**) treatment at the same concentration (Figure 3).

Since P21 expression can be upregulated in a P53-dependent or P53-independent manner [28], we investigated the P53 expression and activation level. Immunoblots, carried out at 48 and 72 h after the administration of either compound **7** or compound **4**, showed a significant increase in P53 protein expression (Figure 3). The pSer15-P53 (pP53) expression level was higher in salviolone-treated A375 cells in comparison with that in untreated samples (Figure 3). Conversely, no significant increase in the P53 phosphorylation level was detected with cryptotanshinone treatment at the same concentration (Figure 3).

Data suggest that after 48 and 72 h of treatment, salviolone more than cryptotanshinone could be able to induce the expression of P21 by stabilizing the tumor-suppressor P53 protein amount as well as by activating its phosphorylation.

#### 2.5.2. Salviolone and Cryptotanshinone Affect Pro-Survival Pathways in the A375 Cell Line

The STAT3 pathway is an important target for therapy in the majority of tumors, and it is hyper-activated in cells and tissue samples from melanoma [29]. Therefore, we investigated whether salviolone (**7**) and cryptotanshinone (**4**) can affect STAT3 activation.

A prerequisite for STAT3 transcriptional activity is its phosphorylation. The phosphorylation on tyrosine 705 is a crucial event triggering the transcription of many STAT3 target genes, although another phosphorylation site is the serine 727 residue [30].

**Figure 4:**
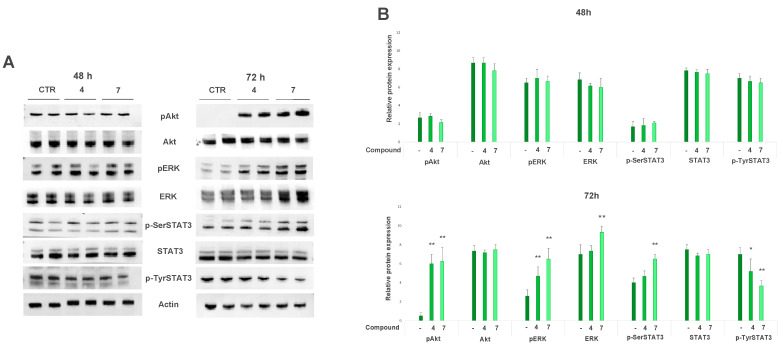
Cryptotanshinone (**4**) and salviolone (**7**) effect on protein expression involved in pro-survival signaling. A375 melanoma cells were cultured in the presence (20 µM) or absence of cryptotanshinone (**4**) or salviolone (**7**) for 48 and 72 h. Immunoblots show the expression levels of (**A**) the activated and total forms of the signal transducer and activator of transcription (STAT)3, the extracellular signal-regulated kinases (ERK)1/2 and the protein kinase B (Akt); (**B**) histograms show the mean values ± SD of the protein expression level measured after 48 or 72 h deriving from three to four independent experiments. All comparisons were performed vs. each control sample after normalization with *β*-actin expression. * *p* < 0.05; ** *p* < 0.01.

An immunoblot performed after 72-h culture in the presence of either compound **7** (20 µM) or compound **4** (20 µM) showed a significant reduction in Tyr705-STAT3 phosphorylation in comparison to untreated cells (Figure 4). Unexpectedly, the p-Ser727-STAT3 level showed an increase after 72-h treatment (Figure 4).

Several serine-threonine kinases can be responsible for Ser727-STAT3 phosphorylation [31], among them, extracellular-signal-regulated kinases (ERK1/2), and the phosphoinositide-3-kinase/protein kinase B (Akt)/mechanistic target of rapamycin (mTOR) pathways has a key role [31,32]. In A375 cells cultured for 48 h, STAT3 was constitutively activated by the presence of both tyrosine and serine phosphorylation, but it did not significantly change its activity with both treatments. Likewise, ERK1/2 and Akt were in their phosphorylated and activated states in all samples (treated and control). Instead, after 72-h culture, the expression level of pERK1/2 and pAkt declined in the control sample, whereas it remained high in A375 cells treated with either salviolone (**7**) or cryptotanshinone (**4**) (Figure 4). It must be noted that although the expression level of Akt total protein was not changed with both treatments after 48 and 72 h, the total protein amount of ERK1/2 increased only with salviolone after 72-h treatment parallel to the increase in its phosphorylated form (Figure 4).

### 2.6. Effect of MEK1-Inhibitor U0126 and PI3K-Inhibitor LY294002 on A375 Cells Treated with Salviolone or Cryptotanshinone

**Figure 5:**
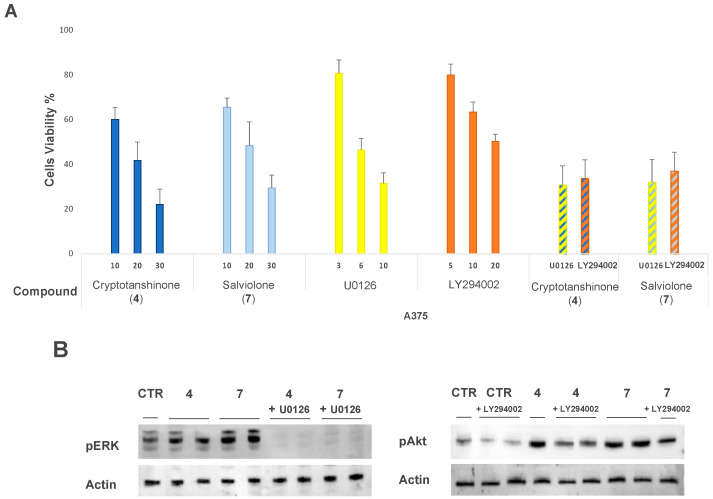
Cell viability of salviolone or cryptotanshinone treatment in the presence or absence of ERK1/2 or Akt inhibitors. (**A**) SRB assay compares the effects on A375 cell viability of 72-h treatment with salviolone (**7**) (light blue) or cryptotanshinone (**4**) (blue) alone, U0126 (yellow) or LY294002 (orange) alone, and compound **7** or **4** (20 µM) plus ERK1/2- or Akt-inhibitor (10 µM) co-administration (striped yellow and striped orange, respectively). (**B** left) Immunoblot attests the inhibitory effect of U0126 (10 µM) on ERK1/2 activation in a co-treatment with 20 µM cryptotanshinone (**4**) or salviolone (**7**). (B, right) Immunoblot attests the inhibitory effect of LY294002 (10 µM) on Akt activation in a co-treatment with 20 µM cryptotanshinone (**4**) or salviolone (**7**). Data acquired calculating the average ± SD of values obtained from three to four independent experiments were compared to data from non-treated control.

We demonstrated that salviolone (**7**) or cryptotanshinone (**4**) elicits the phosphorylation of ERK1/2 and Akt in A375 cells. Thus, we verified the effect of ERK1/2 or Akt activity on cell growth. In this regard, we blocked ERK1/2 activation using 10 µM U0126, a selective inhibitor of the ERK-upstream kinase MEK1 [33]. Moreover, we hindered Akt activation with 10 µM LY294002, an inhibitor of PI3K [34]. In addition, 72-h culture of A375 cells in the presence of U0126 or LY294002 administered alone inhibited A375 cell viability in a concentration-dependent manner (Figure 5A). However, the administration of 10 µM U0126 or LY294002 to the culture medium 2 h before either 20 µM salviolone (**7**) or cryptotanshinone (**4**) addition did not potentiate the decrease of A375 cell viability induced by the two diterpene compounds (Figure 5A). As expected, immunoblot data attested that 10 µM U0126 co-administered with either compound **4** or compound **7** at 20 µM strongly inhibited the expression level of pERK1/2 (Figure 5B). Likewise, 10 µM LY294002 co-administration with compound **7** or **4** at 20 µM induced an inhibition of the pAkt expression level (Figure 5B). Data suggest that the persistent activation of ERK1/2 and Akt, elicited by salviolone (**7**) or cryptotanshinone (**4**), does not represent a pro-survival signaling for the A375 cell line.

### 2.7. Salviolone and Cryptotanshinone Inhibit the Ability of A375 Cells to Form Colonies in Soft Agar

**Figure 6:**
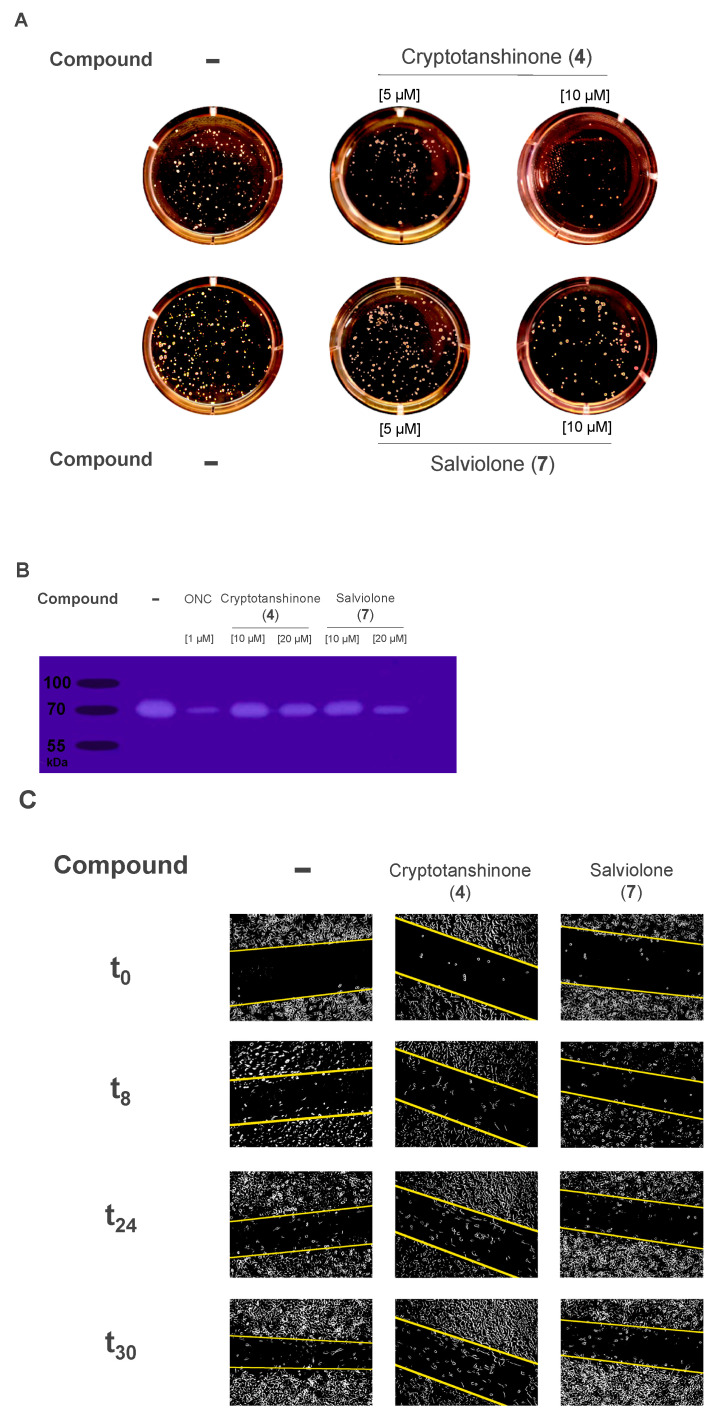
Cryptotanshinone (**4**) and salviolone (**7**) effect on colony formation, digestion of extracellular matrix, and migration. (**A**) A colony formation assay of A375 melanoma cells with 5 and 10 µM cryptotanshinone (**4**) or salviolone (**7**) cultured for 21 days in soft agar. (**B**) A gel zymography assay showing the ability of cryptotanshinone (**4**) or salviolone (**7**) to inhibit the metalloproteinase (MMP)2 gelatinase activity in A375 cells. (**C**) A wound healing assay attesting the inhibition of cell mobility with 20 µM cryptotanshinone (**4**) in the A375 cell line. Images were captured with Zeiss Axio Vert (10×) at time 0, 8, 24, and 30 h after the administration of each compound. For A, B, and C, the images reported a representative picture of three independent experiments.

To investigate the salviolone (**7**) and cryptotanshinone (**4**) capability to inhibit melanoma cell growth in an anchorage-independent manner, a soft agar colony formation assay was performed. After 21-day culture, either salviolone (**7**) or cryptotanshinone (**4**) at 5 and 10 µM decreased the number and dimension of colonies formed on soft agar (Figure 6A).

### 2.8. Salviolone and Cryptotanshinone Inhibit Matrix Metalloproteinase 2 (MMP2) Activity in A375 Cells

A gel zymography assay can measure the ability of matrix metalloproteinases (MMPs) to digest their substrate gelatin present in the gel. Thus, the zymographic approach allows sensitive and quantifiable analysis of the activity of MMPs that can be recognized by their molecular weight [35]. Gel zymography data attested that MMP2 is the most active gelatinase present in A375 control cells (Figure 4B). Both compounds **7** and **4** at 10 and 20 µM inhibited MMP2 gelatinase activity in the A375 melanoma cell line (Figure 6B).

### 2.9. Cryptotanshinone but Not Salviolone Inhibits A375 Cell Migration 

The wound healing assay is widely used to measure cell migration. The method is based on quantifying the rate at which cells can close an artificial scratch created in a confluent cell monolayer.

Figure 6C shows the ability of 20 µM cryptotanshinone (**4**) to slow down the migration rate of A375 cells. Instead, in comparison to controls, the presence in the cell culture medium of salviolone (**7**) did not significantly delay the wound healing closure (Figure 6C).

## 3. Discussion

The phytochemical investigation of *S. miltiorrhiza* roots allowed us to isolate seven compounds (**1**–**7**) belonging to the diterpene class. Their structures have been characterized based on NMR experiments along with MS analysis. Tanshinone IIA (**1**), 1α-hydroxytanshinone (**2**), 1-oxotanshinone (**3**), cryptotanshinone (**4**), and 1*β*-hydroxycryptotanshinone (**5**) represent tanshinone derivatives characterized by an ortho-quinone system on the tetrahydrophenantrene core and different oxidations at C-1 of the A ring. The D ring is a furan in compounds **1**–**3** and a dihydrofuran in compounds **4** and **5**. Chemically, in 1α-hydroxyanhydride-16*R*-cryptotanshinone (**6**) ortho-quinone, the tetrahydrophenantrene moiety is modified to be an anhydride group. Salviolone (**7**) is a rare bisnorditerpene with a benzotropolone chromophore. It represents the first tropolone-type compound isolated from *S. miltiorrhiza* [36].

With the aim to quantify secondary metabolites isolated in the ethanol extract of *S. miltiorrhiza* roots, a quantitative determination by LC-ESI/QTrap/MS system, in positive ion mode, was carried out. LC-ESI/Qtrap/MS analysis using a multiple reaction monitoring (MRM) experiment is considered one of the most suitable techniques for the quantification of metabolites. In agreement with literature data, tanshinone IIA (**1**) and cryptotanshinone (**4**) exhibit the highest concentrations in *S. miltiorrhiza* roots, followed by 1-oxotanshinone (**3**) (Table 1); therefore, they represent the chemical markers of *S. miltiorrhiza* roots [37]. 1*α*-hydroxytanshinone (**2**), 1α-hydroxyanhydride-16*R*-cryptotanshinone (**6**), and salviolone (**7**) occurred in lower amounts; 1*β*-hydroxycryptotanshinone (**5**) was not quantified, since its amount in the ethanol extract of *S. miltiorrhiza* roots was found to be below the LOD value of the applied method.

Melanoma is a highly malignant solid tumor characterized by an elevated growth and propagation rate [1]. Surgical removal and chemotherapy are the conventional treatments. However, these treatments cannot often prevent recurrences or the appearance of metastasis. Therefore, to eradicate the disease, new anti-melanoma agents exhibiting low toxicity in normal cells should be discovered.

To this aim, we tested diterpene compounds **1**–**7** on two human melanoma cell lines concerning their ability to inhibit malignant cell growth.

Even though the highly active compounds tanshinone IIA (**1**) and cryptotanshinone (**4**) are also the most representative compounds in terms of amount, in this work, the bioactivity profiles of the less explored compounds **2**–**3** and **5**–**7** have been investigated. Our results show the ability of the benzotropolone derivative salviolone (**7**) to inhibit A375 and MeWo cell viability with effectiveness comparable to that of cryptotanshinone (**4**). Although showing a different skeleton, salviolone (**7**) has a common structural feature with tanshinone IIA (**1**) and cryptotanshinone (**4**), which is the absence of the oxygenated function at C-1 of ring A, occurring in compounds **2**–**3** and **5**–**6,** which were almost inactive. Thus, this characteristic seems to be crucial for the investigated activity.

Therefore, we decided to better explore the anti-melanoma activity of salviolone since no reports are so far available on its effectiveness in melanoma cells.

Remarkably, treatment with either salviolone (**7**) or cryptotanshinone (**4**) at 20 µM for 72 h showed about 50% inhibition of A375 and MeWo melanoma cell viability (Figure 2). However, salviolone (**7**) and cryptotanshinone (**4**) administration proved to be safe for culture of normal human epithelial melanocytes (NHEM) because 72-h treatment with these compounds at 40–60 µM did not affect NHEM growth (Figure 2). Data suggest that compounds **7** and **4** could display elevated specificity to block the highly proliferating malignant cell growth while preserving at the same time normal cell viability.

Among the two melanoma cell lines used in this study, A375 is the most highly malignant because it displays low time of duplication, shows the activation of multiple oncogenic pathways, and carries a high pathogenic mutation in the BRAF gene [38,39]. Therefore, we chose A375 cells to investigate the molecular mechanisms underlining the growth inhibition induced by salviolone administration in comparison to that by cryptotanshinone using the same concentrations for each compound.

Cell cycle deregulation is a hallmark of cancer, resulting in accelerated cell duplication, for which cell-cycle-related proteins are sensible targets for tumor therapy.

Progression of the cell cycle is a tightly regulated process that involves the sequential activation and inhibition of Cdks [40] and hyper-phosphorylation of Rb, which induces cyclins expression, allowing the cells to advance in the cell cycle [40].

In A375 cells, 48- and 72-h treatment with salviolone (**7**) or cryptotanshinone (**4**) induced a significant increase in both P53 and P21(Cip1/Waf1) expression levels (Figure 3). In addition, salviolone but not cryptotanshinone was able to enhance the phosphorylation level of P53, increasing the tumor suppressor activity of the protein (Figure 3). All these effects can mediate a P53-dependent A375 growth inhibition. Accordingly, high levels of P53 and/or P21 protein expression have been found in cryptotanshinone-treated culture of cell lines derived from tongue squamous carcinoma [17], hepatocellular carcinoma [41], and mouse melanoma [19]. Salviolone’s impact on P53 and P21 in any tumor model is unknown since the anticancer effect of salviolone is poorly investigated. We firstly report here salviolone’s capability to affect human melanoma cell growth and its competence to increase P21 and P53 protein expression and activity.

To explore the targets of p53 and p21 activity, we analyzed the expression or activation of some cell-cycle-related proteins in A375 cells treated with salviolone (**7**) and cryptotanshinone (**4**) in comparison with that in not-treated control ones. Our data showed that salviolone and, to a lesser extent, cryptotanshinone were able to decrease Rb phosphorylation, Cdk2 activation, and cyclin A2 expression at both 48 and 72 h (Figure 3).

Our data demonstrated a multitarget activity of salviolone (**7**) and cryptotanshinone (**4**) on cell-cycle-related proteins. This multiple regulation is not surprising since it is mediated by the high expression of P21, which can inhibit several Cdks involved in either the G1 or the S phase of the cell cycle [26]. Indeed, the low expression level of cyclin A2 found by us in A375 cells treated with salviolone (**7**) is consistent with the results of Zhang et al. [42], who previously reported the salviolone potential to arrest G2/M transition in the HeLa cell line. Furthermore, some papers have demonstrated either G1/S or G2/M cell cycle arrest with cryptotanshinone (**4**) in many cancer cell lines [41,43], including a mouse melanoma cell line [19].

The transcription factor STAT3 has a key role in the onset and progression of cancer. Actually, in a mouse model, the silencing of STAT3 expression results in melanoma growth inhibition [44]. Certainly, the activation of STAT3 elicits the expression of many target genes that are involved in cell proliferation, apoptosis, metastasis, inflammation, immunity, and angiogenesis [45]. STAT3 transcriptional activity is triggered by many different kinases able to phosphorylate its tyrosine 705 and serine 727 residues [46]. STAT3 tyrosine phosphorylation seems to be the most important activating event. Phosphorylation of Tyr705-STAT3 can result from the activity of either non-receptor tyrosine kinases or tyrosine kinase receptors after binding to their specific cytokine or growth factor [46]. However, the biological role of p-Ser727-STAT3 is not completely known. The cross talk between STAT3 phosphorylation at these two different sites is also unclear since p-Ser727 STAT3 can either positively or negatively modulate tyrosine 705 phosphorylation [47,48].

Our data in the A375 cell line attested that 72-h treatment with salviolone (**7**) or cryptotanshinone (**4**) modulated STAT3 activity by decreasing its p-Tyr705 level, although, at the same time, p-Ser727-STAT3 expression was increased (Figure 4). To identify the signaling pathway eliciting this serine phosphorylation increase, the activation status of ERK1/2 and Akt was investigated. The activated forms of these kinases were high at 48 h in all treated and not-treated samples (Figure 4). Conversely, after 72 h, immunoblot data indicated that this activation disappeared in control samples, whereas high levels of pERK1/2 and pAkt, as well as p-Ser727-STAT3, were maintained in cells treated with salviolone (**7**) or cryptotanshinone (**4**) (Figure 4). These results, finally, suggest that the activation of ERK1/2 and Akt is a transient occurrence in not-treated samples but a long-lasting event in A375 cells treated with compounds **4** and **7**.

In this regard, a negative correlation has been previously found between ERK1/2 activation and the p-Tyr705-STAT3 expression level in pancreatic cancer cells [49]; in addition, ERK or PI3K, the upstream kinase of Akt, can inhibit STAT3 tyrosine phosphorylation in human melanoma cells [50]. Moreover, it has been previously reported in melanoma cells that MEK-inhibitor resistance could also depend on the cross talk between ERK1/2 and Src/Fak/STAT3 signaling pathways [51], although this topic is still debated and context dependent. Usually, the reactivation of ERK1/2 and/or Akt in cancer cells could indicate an adaptation of cells to the cytostatic compound leading to the onset of resistance. In patients with BRAF-mutated melanoma, MEK inhibitors are more frequently used in association with BRAF inhibitors to definitely block ERK activity and the associated cell proliferation [52]. Unfortunately, these treatments do not have long-lasting effectiveness because drug resistance occurs soon [53].

To investigate the role of ERK1/2 and Akt activation, we tested whether the addition of ERK1/2 or Akt inhibitors can affect A375 cell viability in the presence or absence of salviolone (**7**) or cryptotanshinone (**4**). Firstly, we attested by an immunoblot that U0126 and LY294002 inhibitors were able to decrease the phosphorylation levels of ERK1/2 and Akt, respectively (Figure 5B). An SRB viability assay demonstrated that either U0126 or LY294002 administered alone decreases A375 cell viability in a concentration-dependent manner, suggesting that the transient ERK1/2 or Akt inhibition led to a slowing rate of cell proliferation. Conversely, co-treatment with each inhibitor at 10 µM plus either compound **7** or **4** at 20 µM has not registered additive effects on cell viability in comparison with each single treatment (Figure 5A). Data suggest that the salviolone- and cryptotanshinone-induced sustained activation of ERK1/2 and Akt could be irrelevant regarding the effect elicited by the two diterpene compounds on A375 cell growth. Alternatively, we could suggest that the inhibition of the p-Tyr705-STAT3 level may be the predominant occurrence to slowdown A375 cell proliferation. Furthermore, it should be recalled that the activation of ERK1/2 does not always lead to an increase in cell proliferation. Tsukada et al. indeed have reported in the HepG2 cell line that a strong and long-lasting activation of ERK1/2 results in a paradoxical effect with Rb hypo-phosphorylation, cell cycle arrest, and proliferation inhibition [54].

Besides cell growth inhibition, salviolone (**7**) and cryptotanshinone (**4**) could counteract other important features of A375 melanoma cells related to their high grade of malignancy and ability to metastasize.

Tumor cells can grow in an anchorage-independent manner. Eradication of this capability is required for the prevention of recurrences [55]. Therefore, we measured both salviolone (**7**) and cryptotanshinone (**4**) competence to inhibit A375 colony formation in soft agar. We found that 5–10 µM salviolone (**7**) and cryptotanshinone (**4**) reduced the number of colonies present in soft agar in a concentration-dependent manner (Figure 6A), thus, suggesting that both compounds can inhibit an important feature of malignant cells, i.e., their ability to grow and divide irrespective of their extracellular matrix contact.

In this regard, salviolone (**7**) and cryptotanshinone (**4**) can also inhibit the gelatinase activity of MMP2 in a concentration-dependent modality (Figure 6B). MMPs are proenzymes and need prior activation to be active. The zymography assay used by us can measure only the active forms of MMPs, which are correlated with the potential invasiveness of melanoma cells. Indeed, MMPs can cleave collagen, elastin, and other extracellular matrix components favoring cancer cell spread [56]. Moreover, MMP2 is a direct target gene of STAT3 and both STAT3- and MMP2-activation has been reported to increase the chances of metastasis [57].

The wound healing assay is a standard in vitro technique for probing the collective cell migration rate of closure of an artificial scratch, which is a measure of the speed of motion of the cells. Salviolone (**7**) was not able to significantly slow down the A375 cell migration rate in comparison to not-treated cells. Instead, as previously reported by Ye et al. [20], cryptotanshinone (**4**) can significantly slow down the A375 cell migration rate. The migration of cells is due to a multitude of signals and mechanisms that are involved in cancer progression and metastasis. At present, we have not identified the mechanism underlying the different competence levels of the two diterpenes to attenuate cell migration.

## 4. Materials and Methods

### 4.1. General Procedures

NMR experiments were acquired in methanol-d4 (99.95%, Sigma-Aldrich, Milan, Italy) on a Bruker DRX-600 spectrometer (Bruker BioSpin GmBH, Rheinstetten, Germany) equipped with a Bruker 5 mm TCI CryoProbe at 300 K. Data processing were carried out with Topspin 3.2 software (Bruker BioSpin, Rheinstetten, Germany).

### 4.2. Plant Material

The powdered roots of *S. miltiorrhiza* Bunge were purchased by Lymeherbs. On the package, the batch number (SM223030), the expiration data (02/2023), and the condition of storage at room temperature in a dry place are reported. A voucher specimen (n. 157) was deposited at the Department of Pharmacy, University of Salerno.

### 4.3. Extraction and Isolation

The powdered roots of *S. miltiorrhiza* (100 g) were milled and macerated at room temperature with ethanol (3 × 0.8 L). After filtration and evaporation of the solvent to dryness in vacuo, 7.88 g of a crude ethanol extract were obtained. The dried ethanol extract (3.0 g) was fractionated on a Sephadex LH-20 (Pharmacia) column (100 × 5 cm), using MeOH as the mobile phase, affording 60 fractions (8 mL), monitored by TLC.

Fractions 8–29 were subjected to an RP-HPLC-UV system with a Synergi Hydro RP 80A (250 × 10 mm). The elution gradient was obtained using water with 0.1% formic acid as eluent A and acetonitrile with 0.1% formic acid as B at a flow rate of 2.0 mL/min; the detection wavelength was 280 nm, and the analysis were performed at room temperature. The HPLC gradient was 40% B for 5 min; from 40 to 80% B for 35 min; from 80 to 90% for 5 min; from 90 to 100% B for 5 min; and, at the end, 100% B for 10 min; in particular, fractions 8–13 (50.5 mg) were chromatographed to obtain compound **7** (5.1 mg; *t_R_* = 22.35 min). Fractions 8–13 (50.5 mg) were chromatographed to obtain compound **7** (5.1 mg; *t_R_* = 22.35 min). Fractions 23–24 (65.3 mg) gave compound **5** (3.6 mg; *t_R_* = 16.74 min). Fractions 27–29 (100.1 mg) were chromatographed to yield compounds **2** (4.2 mg; *t_R_* = 23.03 min) and **3** (3.5 mg; *t_R_* = 15.07 min). Fractions 30-36 (220.6 mg) were chromatographed to yield compounds **4** (21.5 mg; *t_R_* = 18.57 min) and **1** (24.3 mg; *t_R_* = 21.52 min).

Fractions 37–39 (62.2 mg) were chromatographed by semipreparative HPLC-RI with a Waters XTerra Prep MS C18 column (300 × 7.8 mm i.d.) using MeOH-H_2_O (61:39) as the mobile phase to yield compound **6** (3.8 mg; *t_R_* = 14.00 min).

To unambiguously identify compounds isolated by HPLC and to discriminate among structural isomers or stereoisomers, structure elucidation by 1D and 2D NMR experiments of isolated compounds was carried out. The purity of these compounds (>99%) was determined by HPLC analysis.

### 4.4. Quantitative Analysis of Compounds **1–7** in S. miltiorrhiza Roots

Quantitative analysis was performed on an LC-ESI/QTrap/MS system, operating in the multiple reaction monitoring (MRM) mode. The MRM approach firstly targets the ion corresponding to the compound of interest, with the subsequent fragmentation of this target ion to produce a range of daughter ions. One (or more) of these fragment daughter ions can be selected for quantitation purposes. The analysis was performed on a C18 reversed phase (RP) column (2.1 × 250 mm; Luna MS C18 5 µm; Phenomenex, Aschaffenburg, Germany) kept at 30 °C, using water with 0.1% formic acid as eluent A and acetonitrile with 0.1% formic acid as B at a flow rate of 0.2 mL/min. The autosampler was set to inject 2 μL of the extract (250 ng/μL).

Stock solutions (1 mg/mL) of isolated compounds used as external standards (ES) were prepared by dissolving each compound in methanol. Each stock solution was diluted with methanol to obtain 10 solutions of different ES concentrations (0.05, 0.5, 0.1, 1.0, 2.5, 5, 10, 12.5, 17.5, and 20 ng/μL). Calibration curves were constructed by injecting 4 μL of each standard solution at each concentration level in triplicate (Table S2). Linear regression analysis was performed using the Analyst 1.6.2 Software provided by the manufacturer (AB Sciex, Milan, Italy).

Linearity was evaluated by correlation values of calibration curves. The limit of quantification (LOQ; equivalent to sensitivity) was evaluated by injecting a series of increasingly diluted standard solutions until the signal-to-noise ratio was reduced to 10. The limit of detection (LOD) was estimated by injecting a series of increasingly diluted standard solutions until the signal-to-noise ratio was reduced to 3 [58]. The LOD was from 0.035 to 0.086 µg/µL and the LOQ from 0.15 to 0.38 µg/µL.

### 4.5. Malignant Melanoma and Normal Melanocyte Cell Culture

Human MeWo (HTB-65) and A375 (CRL-1619) melanoma cell lines as well as the normal human epidermal melanocyte (NHEM) (PCS-200-013) cell line (ATCC, Manassas, VA, USA) were cultured at 37 °C under 5% CO_2_ in a humidified atmosphere. Roswell Park Memorial Institute 1640 medium (RPMI, Gibco, Ref.21875, Thermo Scientific, Waltham, MA, USA) was used to culture MeWo cells, while Dulbecco’s Modified Eagle Medium (DMEM, Gibco, Ref. 61965, Thermo Scientific, Waltham, MA, USA) was used for A375 and NHEM cell lines. The culture media were supplemented with 10% fetal bovine serum (FBS, Gibco, Thermo Scientific, Waltham, MA, USA) plus 1% Antibiotic Antimycotic Solution (Gibco, Thermo Scientific). The MEK1 inhibitor U0126 (CAY-70970) and the PI3K inhibitor LY294002 (CAY-70920) were provided by Cayman Chemical, Ann Arbor, MI, USA.

### 4.6. Cell Viability Sulforhodamine B Assay

Melanoma MeWo and A375 cells or NHEM were seeded in 96-well plates (5.0 × 10^3^ cells/well for MeWo cells and 2.9 × 10^3^ cells/well for A375 or NHEM). After 24-h culture, the cells were treated with different concentrations of compounds **1**–**7**. After 72-h treatment, the cells were fixed by adding 25 µL/well of 50% (*w*/*v*) TCA directly into the culture medium. The plates were incubated at 4 °C for 1 h, washed four times with ddH_2_O, and dried at RT. Staining was performed by adding 50 µL/well of 0.04% (*w*/*v*) sulforhodamine B (SRB) sodium salt solution (Sigma-Aldrich, Milan, Italy). After 1-h incubation at RT, the plates were rinsed with 1% HAc and air-dried. SRB was solubilized in a 10 mM Tris-base solution pH 10.5 and Abs_492_ measured in the plate reader TECAN NanoQuant Infinite M200 Pro (Tecan Group Ltd., Männedorf, Switzerland). Six replicates for each condition/data point were performed.

### 4.7. Total Protein Extracts and Sample Preparation for Immunoblot Analysis

A375 cells were seeded in 6 cm Petri dishes (190 300 cell/dish) and treated with different compounds at 10 or 20 μM. After 48- or 72-h treatment, the cells were scraped using warm 1× sample buffer (2% SDS, 10% glycerol, 50 mM Tris-HCl, 1.75% *β*-mercaptoethanol, and bromophenol blue) and boiled at 99 °C for 10 min. Total protein extracts were kept at −80 °C until use.

### 4.8. Immunoblot Analysis

Protein extracts were analyzed with a 7.5 or 10% polyacrylamide SDS-PAGE and transferred to a polyvinylidene difluoride membrane (PVDF, Thermo Fisher Scientific, Waltham, MA, USA). The membranes were blocked at RT for 1 h with TBST buffer (10 mM Tris-HCl pH 7.5, 100 mM NaCl, 0.1% Tween20) containing 4% Milk (Formulat). Then, they were incubated on a shaker, overnight (ON) at 4 °C, with 5% BSA solution containing primary antibodies against p-Tyr705-STAT3 (#9145, 1:1000), pRb (#8516, 1:2000), pERK (#4370, 1:1000), pAkt (#4060, 1:2000), pSer15-P53 (#9286, 1:2000) (Cell Signaling Technology, Danvers, CO, USA), Akt (GTX121937, 1:3000), ERK (GTX134452, 1:6000), Cyclin A2 (GTX-103042, 1:3000), p-Thr160Cdk2 (GTX-133862, 1:3000), p21 (GTX-629543, 1:3000) (Genetex, Alton Pkwj Irvine, CA, USA), p53 (Sc-263, 1:1000), STAT3 (Sc-482, 1:1000), and p-Ser727-STAT3 (Sc-136193, 1:1000) (Santa Cruz Biotechnology, Dallas, TX, USA). The membranes were washed three times with TBST buffer for 30 min and then incubated for 1 h with a horseradish-peroxidase-conjugated secondary antibody (anti-rabbit 1:6000, Genetex), (anti-mouse, 1:4000, Cell Signaling Technology, Danvers, CO, USA). They were washed another three times for 30 min with TBST buffer. All protein extracts were normalized with a β-actin protein antibody (GTX-124214, 1:10,000; Genetex, Alton Pkwj Irvine, CA, USA). Immuno-detection was carried out with an ECL kit (GE-Healthcare, Little Chalfont, UK), and the chemiluminescence signals were detected with ChemiDoc (Bio-Rad, Hercules, CA, USA).

### 4.9. Soft Agar Colony Formation Assay

Before seeding, 6-well plates were prepared with soft agar, as follows: the bottom layer was filled with 1% low-gelling temperature agarose and 2X DMEM, supplemented with 20% FBS and 2% Antibiotic Antimycotic Solution (Gibco, BRL Invitrogen Corp., Carlsbad, CA, USA). After 30 min, cells (5000 cell/well) were suspended in 0.6% agarose and 2X DMEM supplemented with 20% FBS and 2% Antibiotic Antimycotic Solution (Gibco, BRL Invitrogen Corp., Carlsbad, CA, USA) and placed over the 0.5% agarose layer. Then, either cryptotanshinone (**4**) or salviolone (**7**) at 5–10 μM was added directly into each well and 100 μL of fresh media was added twice a week for 3 weeks. The semi-solid cultures were performed in triplicate and images captured.

### 4.10. Gelatin Zymography

A375 cells were cultured in the presence of 10 or 20 µM cryptotanshinone (**4**), salviolone (**7**), or 1 µM onconase (provided by Prof. G. Gotte), as a positive control [59], for 48 h and then they were serum starved for 24 h. The conditioned medium was collected and 10× concentrated with an Amicon Ultra-2 Centrifugal filter unit (Merck-Millipore, Milan, Italy). Protein concentrations were determined with Coomassie (Thermo Fisher Scientific, Waltham, MA, USA) and samples treated with a non-reducing sample buffer. To separate proteins, a 7.5% SDS-PAGE was performed. The gel contained 4 mg/mL of gelatin (Sigma-Aldrich, Milan, Italy) and was washed with 50 mM Tris-HCl pH 7.5, 5 mM CaCl2, and 1 μM ZnCl2 supplemented with 2.5% Triton X-100 (Serva Electrophoresis, Heidelberg, Germany). It was then kept in the incubation buffer (1% Triton X-100, 50 mM Tris-HCl pH 7.5, 5 mM CaCl2, and 1 μM ZnCl2) for 24 h. This buffer contained the cofactors necessary to maintain the metalloproteinase (MMP)2 gelatinase activity, so that it could degrade the gelatin present in the gel. The gel was stained with 0.5% Coomassie Brilliant Blue R-250 in 20% ethanol/10% acetic acid, and the gelatinase activity was detected by evaluating the presence and intensity of clear bands on the blue background.

### 4.11. Wound Closure Cell Migration Assay

The wound healing assay is widely used to measure cell migration. A375 cells were seeded in 12-well plates (120 × 10^3^ cells/well) and, once confluence was reached, the monolayer was scratched with a sterile 10 μL pipette tip. To remove detached cells, the wells were washed with the complete medium and afterward refilled with a fresh medium containing 0.5% FBS. Then, either cryptotanshinone (**4**) or salviolone (**7**) at 10 or 20 µM was added to the culture. After 0-, 8-, 24- and 30-h culture, images were captured with Zeiss Axio Vert. (Zeiss, Milan, Italy) to measure the delay to repopulate the scratch in comparison with the non-treated control sample.

### 4.12. Statistics

All the results are reported as a mean value ± SD. Unless otherwise noted, the *p*-values were determined using unpaired, two-tailed Student’s *t*-test, with one asterisk * if *p* < 0.05 and two ** if *p* < 0.01. For each type of experiment, a minimum of three independent biological replicates were performed.

## 5. Conclusions

We purified seven natural diterpenoid derivatives from *Salvia miltiorrhiza* roots, and we analyzed their anti-melanoma activities. Besides the most known anti-melanoma effect of tanshinone IIA (**1**) and cryptotanshinone (**4**), we firstly showed that only salviolone (**7**), among the diterpene compounds, displays high competence to inhibit A375 and MeWo melanoma cell growth without affecting normal melanocyte viability. In A375 cells, the molecular mechanisms of both salviolone (**7**) and, to a lesser extent, cryptotanshinone (**4**) pass through a cell cycle hindered by the over-expression of P21 in a P53-dependent manner. This effect results in slowing down cell cycle progression in a multitarget action, including pRb, pCdk2, and cyclin A2 downregulation. Salviolone (**7**) and cryptotanshinone (**4**) were also able to decrease the p-Tyr-STAT3 expression level, which can justify the reduction of both MMP2 gelatinase activity and soft agar colony formation, thus lowering A375 malignancy potential. At the same time, salviolone (**7**) and cryptotanshinone (**4**) can induce the sustained activation of ERK1/2 and Akt without decreasing their growth-inhibitory effectiveness.

## Figures and Tables

**Table 1:** Quantitative results of compounds **1**–**7** occurring in the EtOH extract of *S. miltiorrhiza* roots.

Compound	MRM Transition	R^2^	Regression Line	mg/100 g Roots ± SD
Tanshinone IIA (**1**)	295 → 277	0.992	y = 3.50 × 10^3^x + 1.15 × 10^6^	1361.51 ± 33.39
1α-Hydroxytanshinone (**2**)	311 → 293	0.995	y = 103x − 4.91 × 10^4^	83.00 ± 1.82
1-oxotanshinone (**3**)	309 → 281	0.996	y = 170x − 5.12 × 10^4^	353.65 ± 11.08
Cryptotanshinone (**4**)	297 → 251	0.995	y = 4.75 × 10^3^x + 2.30 × 10^6^	1379.00 ± 55.72
1*β*-Hydroxycryptotanshinone (**5**)	313 → 267	0.994	y = 2.72 × 10^3^x + 6.63 × 10^5^	N.D.
1α-Hydroxyanhydride-16*R*-cryptotanshinone (**6**)	329 → 267	0.991	y = 1.73 × 10^3^x + 2.26 × 10^4^	20.44 ± 0.89
Salviolone (**7**)	269 → 251	0.992	y = 96.2x + 3.675 × 10^4^	31.60 ± 0.78

## Data Availability

Not applicable.

## Supplementary Materials

The following are available online at https://www.mdpi.com/article/10.3390/ijms23031121/s1.
