# Supplementary material for: Salviolone from Salvia miltiorrhiza Roots Impairs Cell Cycle Progression, Colony Formation, and Metalloproteinase-2 Activity in A375 Melanoma Cells: Involvement of P21(Cip1/Waf1) Expression and STAT3 Phosphorylation
Source: Int J Mol Sci. 2022 Jan 20;23(3):1121. doi: 10.3390/ijms23031121 (PMC8835475; doi:10.3390/ijms23031121)
Supplement: Supplementary file 1 [file ijms-23-01121-s001.zip › ijms-1547168-supplementary.pdf]

Supporting information

Figure S1. ^{1}H NMR Spectrum (600 MHz, CD_3OD) of compound **1**.

Figure S2. ^{1}H NMR Spectrum (600 MHz, CD_3OD) of compound **2**.

Figure S3. ^{1}H NMR Spectrum (600 MHz, CD_3OD) of compound **3**.

Figure S4. ^{1}H NMR Spectrum (600 MHz, CD_3OD) of compound **4**.

Figure S5. ^{1}H NMR Spectrum (600 MHz, CD_3OD) of compound **5**.

Figure S6. ^{1}H NMR Spectrum (600 MHz, CD_3OD) of compound **6**.

Figure S7. ^{1}H NMR Spectrum (600 MHz, CD_3OD) of compound **7**.

Table S1. ^{13}CNMR Data (150 MHz, CD_3OD, δ ppm) of compounds **1**-**7**.

Table S2. LC–MS/MS conditions for quantitation of compounds **1**-**7** by MRM.

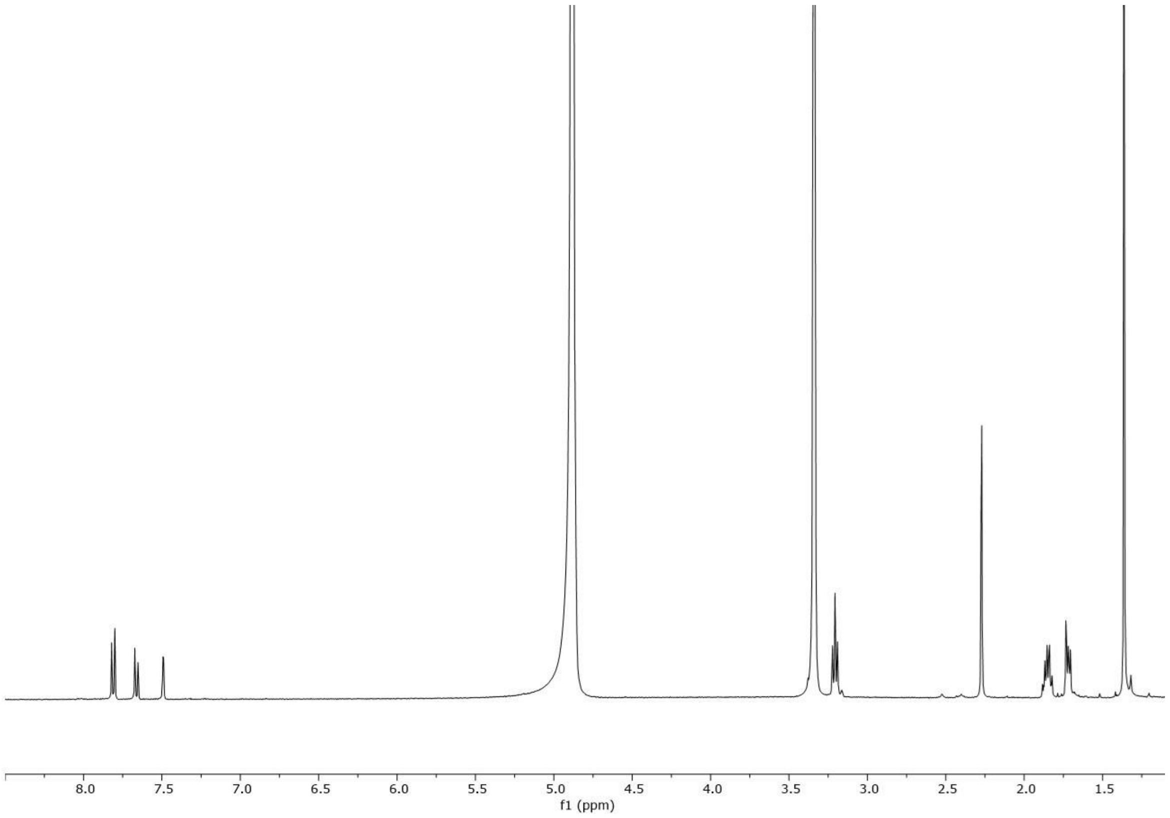

Figure S1. ^{1}H NMR Spectrum (600 MHz, CD_3OD) of compound **1**.

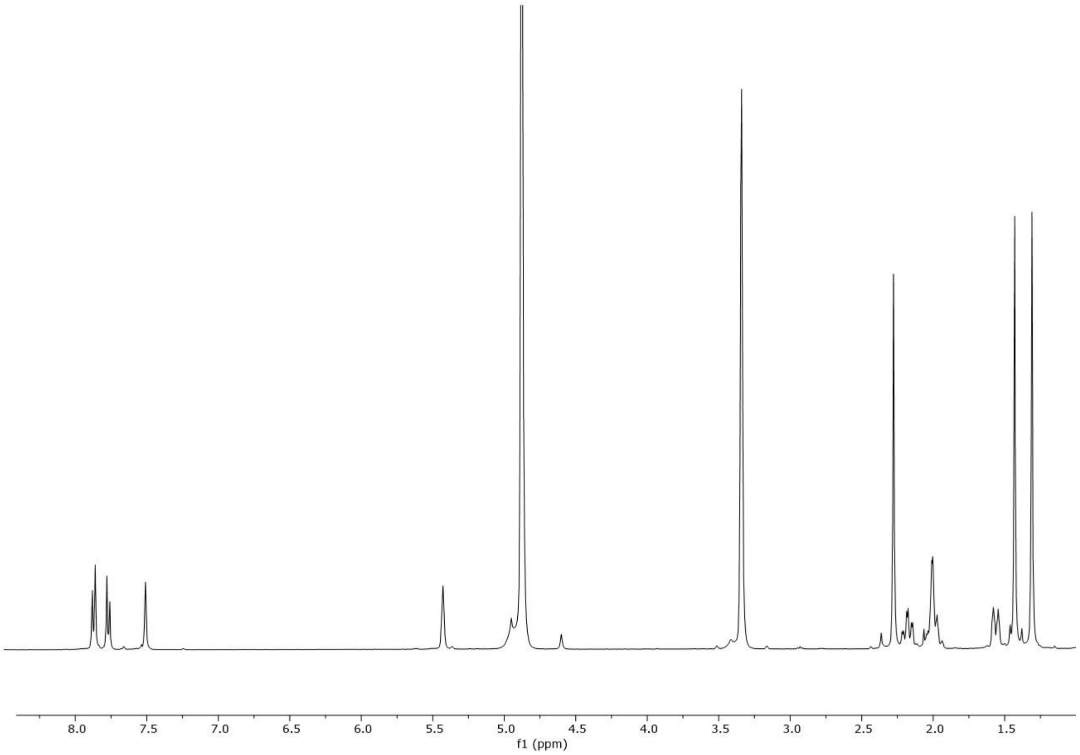

Figure S2. ^{1}H NMR Spectrum (600 MHz, CD_3OD) of compound **2**.

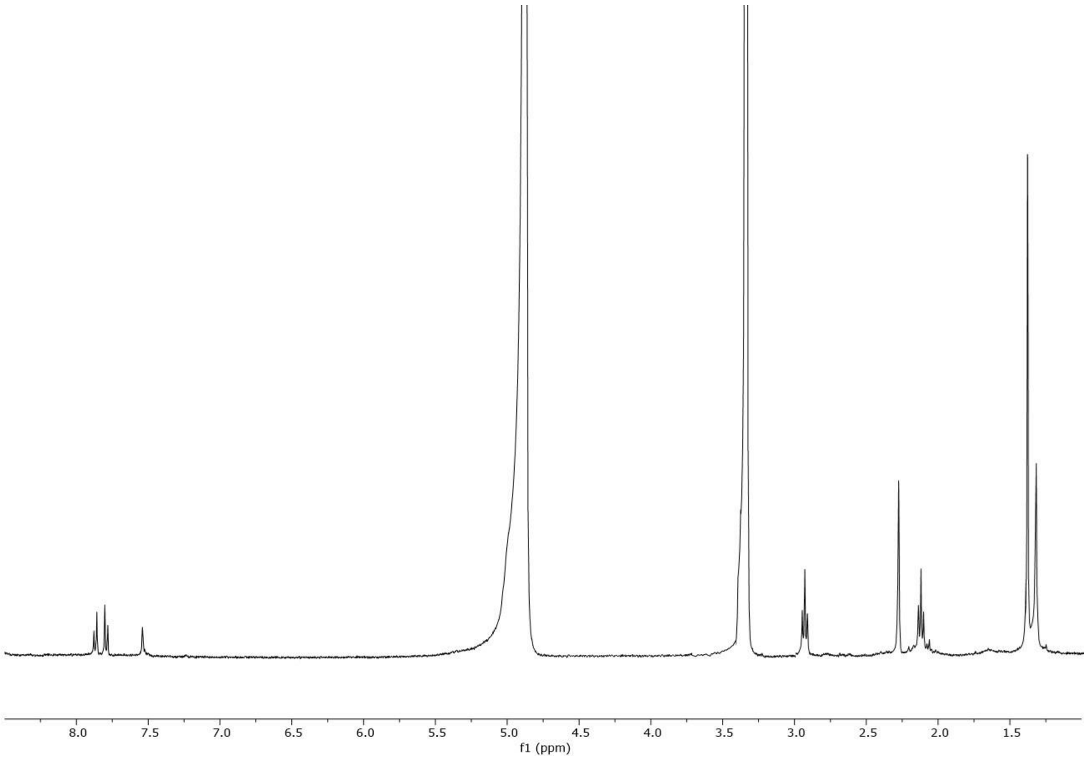

Figure S3. ^{1}H NMR Spectrum (600 MHz, CD_3OD) of compound **3**.

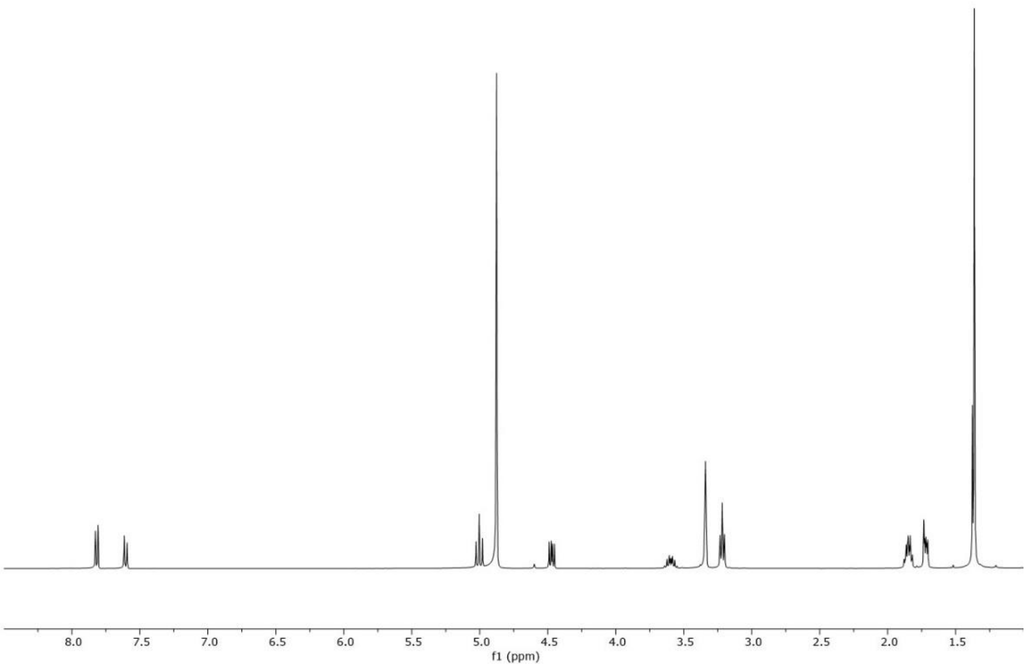

Figure S4. ^{1}H NMR Spectrum (600 MHz, CD_3OD) of compound **4**.

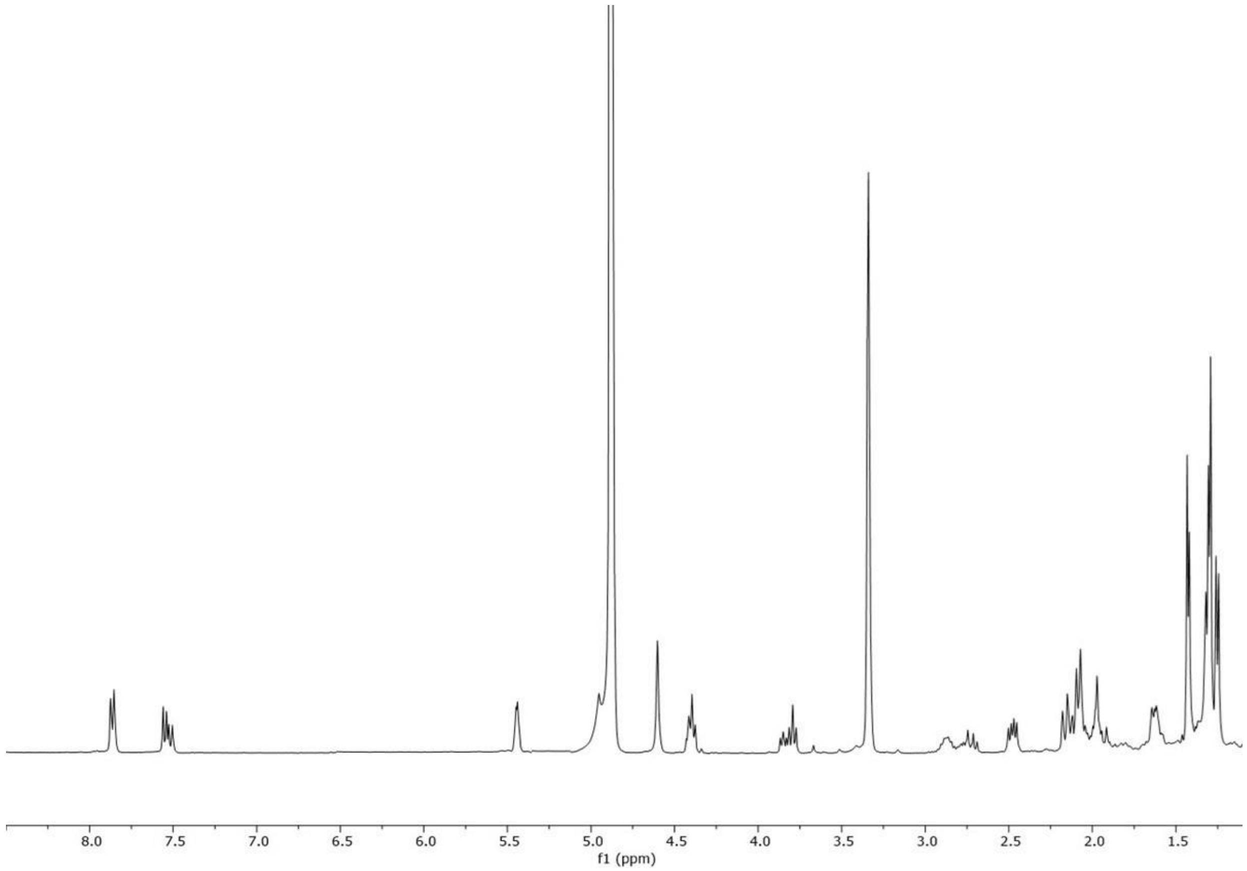

Figure S5. ^{1}H NMR Spectrum (600 MHz, CD_3OD) of compound **5**.

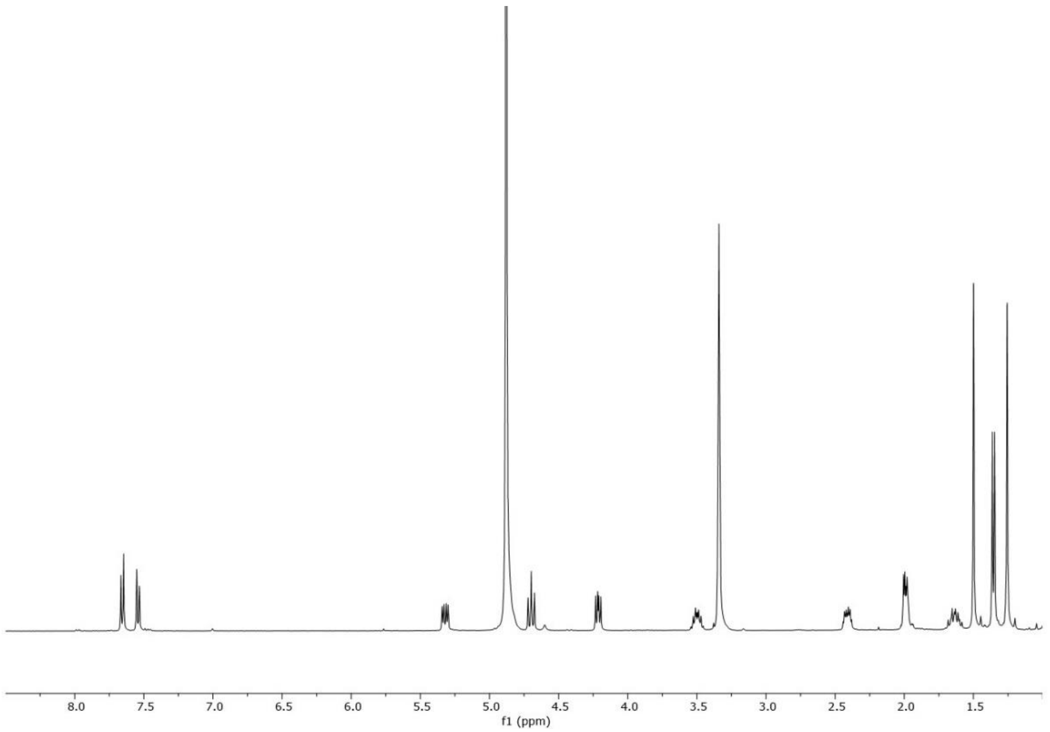

Figure S6. ^{1}H NMR Spectrum (600 MHz, CD_3OD) of compound **6**.

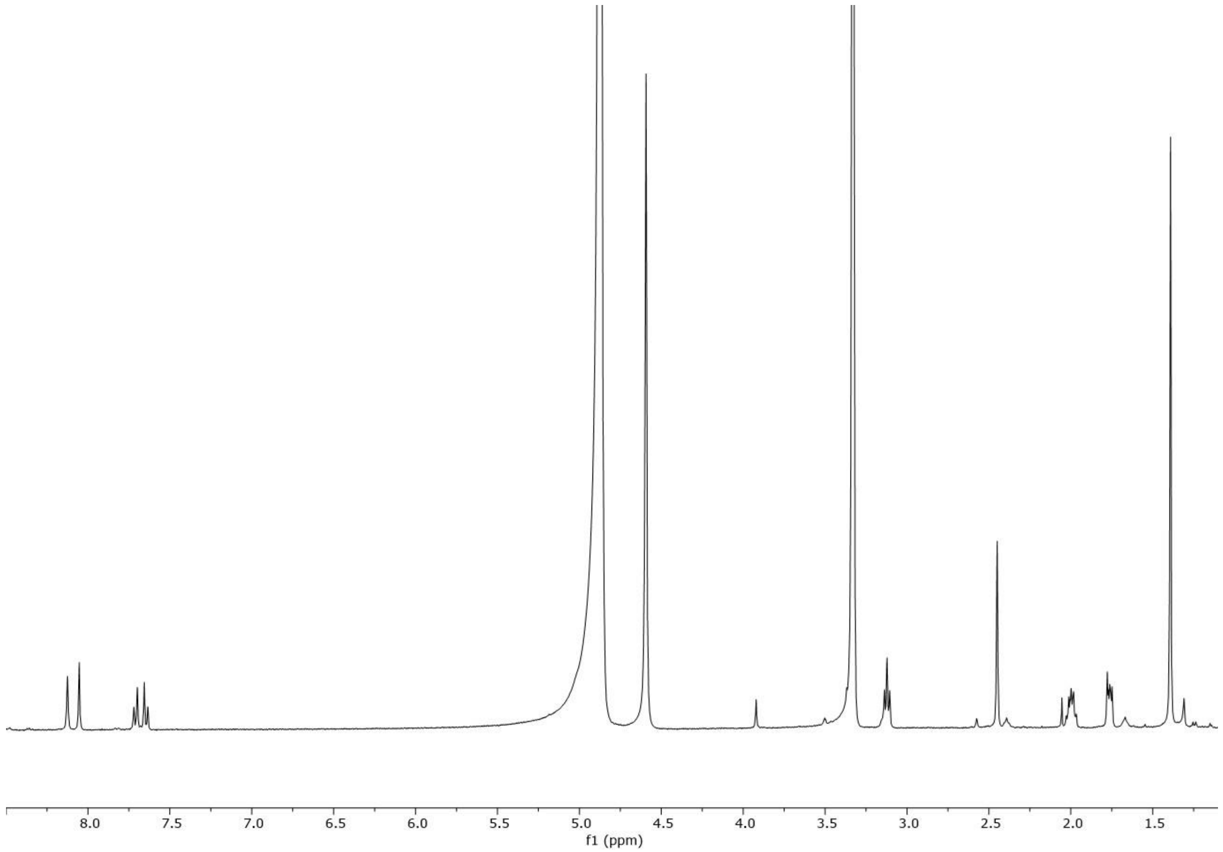

Figure S7. ^{1}H NMR Spectrum (600 MHz, CD_3OD) of compound **7**.

Table S1. ^{13}CNMR Data (150 MHz, CD_3OD, δ ppm) of compounds **1**-**7**.

position	1	2	3	4	5	6	7
1	29.8, CH_2	64.4, CH	200.1, C	30.3, CH_2	63.5, CH	79.0, CH	29.6, CH_2
2	19.3, CH_2	27.9, CH_2	26.9, CH_2	19.8, CH_2	27.0, CH_2	26.7, CH_2	20.4, CH_2
3	38.1, CH_2	32.5, CH_2	37.0, CH_2	38.6, CH_2	32.1, CH_2	37.9, CH_2	38.9, CH_2
4	35.5, C	36.4, C	35.6, C	35.8, C	35.3, C	35.8, C	36.0, C
5	150.3, C	150.7, C	155.7, C	152.7, C	149.7, C	146.0, C	149.2, C
6	134.3, CH	135.8, CH	132.2, CH	133.6, CH	134.8, CH	130.7, CH	127.7, CH
7	123.0, CH	123.1, CH	125.6, CH	123.4, CH	122.8, CH	132.9, CH	132.9, CH
8	127.5, C	146.6, C	129.7, C	126.6, C	138.0, C	128.8, C	134.5, C
9	126.8, C	128.6, C	131.0, C	128.5, C	128.7, C	124.4, C	138.9, C
10	143.9, C	129.3, C	139.0, C	144.1, C	146.7, C	149.1, C	134.9, C
11	185.0, C	185.0, C	186.0, C	185.0, C	185.2, C	170.5, C	113.5, CH

12	175.8, C	176.0, C	175.3, C	176.3, C	176.0, C	168.2, C	155.1, C
13	121.0, C	121.0, C	122.0, C	119.3, C	119.0, C	114.8, C	180.4, C
14	161.5, C	163.2, C	161.4, C	171.5, C	171.0, C	161.0, C	148.7, C
15	141.6, CH	143.4, CH	143.2, CH	82.1, CH_2	77.8, CH_2	79.1, CH_2	144.9, CH
16	121.0, C	121.4, C	123.0, C	34.5, CH	34.7, CH	39.6, CH	22.2, CH_3
17	9.2, CH_3	9.4, CH_3	8.9, CH_3	19.4, CH_3	18.3, CH_3	19.2, CH_3	31.2, CH_3
18	31.8, CH_3	31.9, CH_3	29.0, CH_3	31.8, CH_3	31.5, CH_3	31.9, CH_3	31.2, CH_3
19	31.8, CH_3	33.9, CH_3	29.0, CH_3	31.9, CH_3	31.5, CH_3	31.9, CH_3	-

Table S2. LC–MS/MS conditions for quantitation of compounds **1**-**7** by MRM.

**Compound**	DP	CE	EP	CXP
tanshinone II A (**1**)	+187.0	+26.6	+6.6	+23.8
1α-hydroxy-tanshinone (**2**)	+95.7	+30.0	+6.8	+19.2
1-oxo -tanshinone (**3**)	+110.4	+30.0	+6.9	+12.9
cryptotanshinone (**4**)	+187.9	+29.6	+5.0	+18.7
1β- hydroxy-cryptotanshinone (**5**)	+84.1	+29.6	+7.3	+19.4
1α-hydroxyanhydride-16*R*-cryptotanshinone (**6**)	+113.7	+29.6	+6.6	+15.8
salviolone (**7**)	+111.5	+29.6	+6.9	+14.7

DP, Declustering Potential; **CE**, Collision energy; **EP**, Entrance potential; **CXP**, Collision Cell Exit Potential.
